# Reciprocal regulation of integrin β4 and KLF4 promotes gliomagenesis through maintaining cancer stem cell traits

**DOI:** 10.1186/s13046-019-1034-1

**Published:** 2019-01-18

**Authors:** Binbin Ma, Li Zhang, Yujie Zou, Ruiping He, Qiong Wu, Chuanchun Han, Bo Zhang

**Affiliations:** 1 Department of Neurosurgery, Second Affiliated Hospital, Institute of Cancer Stem Cell, Dalian Medical University, Dalian, 116027 China; 2 Laboratory of Pathogenic Biology, College of Basic Medical Science, Dalian Medical University, Dalian, 116027 China; 3 Nursing Department, First Affiliated Hospital, Dalian Medical University, Dalian, 116011 China; 4 Department of Neurology of Dalian Municipal Central Hospital Affiliated, Dalian Medical University, Dalian, 116033 China

**Keywords:** Glioma stem cells, ITGB4, KLF4, Tumourigenesis

## Abstract

**Background:**

The dismal prognosis of patients with glioma is largely attributed to cancer stem cells that display pivotal roles in tumour initiation, progression, metastasis, resistance to therapy, and relapse. Therefore, understanding how these populations of cells maintain their stem-like properties is critical in developing effective glioma therapeutics.

**Methods:**

RNA sequencing analysis was used to identify genes potentially involved in regulating glioma stem cells (GSCs). Integrin β4 (ITGB4) expression was validated by quantitative real-time PCR (qRT-PCR) and immunohistochemical (IHC) staining. The role of ITGB4 was investigated by flow cytometry, mammosphere formation, transwell, colony formation, and in vivo tumorigenesis assays. The reciprocal regulation between Integrin β4 and KLF4 was investigated by chromatin immunoprecipitation (ChIP), dual-luciferase reporter assay, immunoprecipitation, and in vivo ubiquitylation assays.

**Results:**

In this study, we found that ITGB4 expression was increased in GSCs and human glioma tissues. Upregulation of ITGB4 was correlated with glioma grades. Inhibition of ITGB4 in glioma cells decreased the self-renewal abilities of GSCs and suppressed the malignant behaviours of glioma cells in vitro and in vivo. Further mechanistic studies revealed that KLF4, an important transcription factor, directly binds to the promoter of ITGB4, facilitating its transcription and contributing to increased ITGB4 expression in glioma. Interestingly, this increased expression enabled ITGB4 to bind KLF4, thus attenuating its interaction with its E3 ligase, the von Hippel-Lindau (VHL) protein, which subsequently decreases KLF4 ubiquitination and leads to its accumulation.

**Conclusions:**

Collectively, our data indicate the existence of a positive feedback loop between KLF4 and ITGB4 that promotes GSC self-renewal and gliomagenesis, suggesting that ITGB4 may be a valuable therapeutic target for glioma.

**Electronic supplementary material:**

The online version of this article (10.1186/s13046-019-1034-1) contains supplementary material, which is available to authorized users.

## Background

Glioma is the most common primary malignant brain tumour of the central nervous system. Despite great advances in therapeutic techniques for treating glioma, such as surgery, radiotherapy, and chemotherapy, patients with glioblastoma (GBM) still only have an average survival of 12–15 months [1–4]. Accumulating evidence suggests that glioma are functionally heterogeneous and harbour a subset of tumour cells with stem cell characteristics, including the preferential expression of stem cell markers, enhanced self-renewal ability, and multi-lineage differentiation potential. Those cells are termed glioma stem cells (GSCs) and are highly capable of initiating tumour growth or repopulating tumours after treatment [5–8].

Recently, studies have increasingly demonstrated that GSCs are highly adaptive to various crucial conditions such as nutrient-restricted conditions, hypoxia, or chemo-agent exposure, and actively interact with microenvironmental factors to evade antitumour immune responses, promoting tumour angiogenesis and tumour invasion. Because of these characteristics, GSCs are considered to be responsible for tumour recurrence and the poor outcomes of glioma patients [9–11]. Therefore, investigation of the key regulators involved in maintaining these GSC traits is of great importance to understand glioma progression and to develop novel treatment approaches.

Integrin β4 (ITGB4) also known as CD104 is a laminin-5 receptor which is predominantly expressed in squamous epithelial cells, endothelial cells, immature thymocytes, Schwann cells, and fibroblasts of the peripheral nervous system [12]. In tumours, ITGB4 was first discovered as a tumour-specific antigen. Subsequent studies demonstrated that increased expression levels of ITGB4 were correlated with malignant progression and poor survival rates in squamous cell carcinomas (SCCs) of the skin, lung, head and neck, and cervix [13–15]. Further studies have reported that high expression levels of ITGB4 were found in several types of cancer—including breast, bladder, colon, ovarian, pancreatic, prostate, and thyroid—and were linked to poor prognosis [16–18]. In tumour tissues, the phosphorylation of the cytoplasmic tail of ITGB4 leads to its release from hemidesmosomes and its interaction with growth factor receptors, which promotes the invasion and metastasis of tumour cells [18]. Although ITGB4 has been reported to promote tumourigenesis in many cancers, its role in glioma is still unknown.

Here, we show for the first time that ITGB4 expression is increased in GSC and glioblastoma tissues. Elevated levels of ITGB4 maintained the stem-like properties of GSCs, promoted glioma cell migration and tumorigenesis, and were associated with glioma grades. Further mechanistic studies revealed that KLF4, an important transcription factor, could directly bind to the promoter of ITGB4, facilitating its transcription and contributing to increased ITGB4 expression in glioma. Simultaneously, we found that ITGB4 interacted with KLF4 and decreased its binding to the E3 ligase VHL in glioma cells, which subsequently enhanced KLF4 stability and increased KLF4 expression. Thus, our study reveals that a novel feedback loop exists between KLF4 and ITGB4, which contributes to GSC self-renewal and glioma tumourigenesis.

## Methods

### Cell cultures and reagents

Human glioma cell lines LN229 and U251 were obtained from the American Type Culture Collection (ATCC). LN229 and U251 cells were cultured with DMEM supplemented with 10% foetal bovine serum FBS (ExCell Bio, Lot: FSP500), 2 mM L-glutamine, penicillin (100 U/mL), streptomycin (100 μg/mL) and 0.1% Savelt™ (Hanbio Co. Ltd., 1:1000) in a humidified atmosphere of 5% CO_2_ maintained at 37 °C. The medium was replaced every day, and the cells were passaged before reaching confluence. The following antibodies were used in this study: Nanog (Cell Signaling Technology; #3580), OCT4 (Cell Signaling Technology; #2750), KLF4 (Santa Cruz Biotechnology, SC-20691), KLF4 (Cell Signaling Technology, #12173S), ITGB4 (Proteintech, 21738–1-AP), ITGB4 (Abcam, ab29042), VHL (Cell Signaling; #68547), Beta-actin (Proteintech, 6609–1-lg).

### Flow cytometry

Antibodies for CD133 were purchased from Miltenyi Biotec. Briefly, 10 μL of the antibodies were used to mark 10^6^ cells per 100 μL of buffer for 30 min, in the dark, in a refrigerator (2 °C–8°C), and the marker cells were analysed (BD Accuri C6). For the ALDH1 assay, the ALDH1+ population was detected with an ALDEFLUOR kit (Shanghai Stem Cell Technology Co. Ltd., Shanghai, China) following the manufacturer’s instructions. The ALDH1+ and ITGB4 + cells were analysed by flow cytometry (BD Accuri C6) or sorted (BD FACSCanto II).

### Mammosphere formation assay

Spheres were enriched from LN229 and U251 cells by culturing 200 to 1000 cells/mL in serum-free DMEM-F12 medium (Gibco) supplemented with B27 (1:50, Invitrogen) and 20 ng/mL EGF and bFGF. Nontreated tissue culture flasks were used to reduce cell adherence and support growth as undifferentiated tumour spheres. Cells were cultured for 2 weeks, and the number of spheres with a diameter of more than 100 mm in each well was counted.

### Lentiviral transfection and RNA interference

The lentiviral particles for transduction of shRNA-mediated knockdown of ITGB4 and KLF4 were expressed using pLKO.1 vector. The shRNA sequences used for targeting are as follows: ITGB4, 5´-GAGGGTGTCATCACCATTGAA-3′ (#2) and 5´-CCAGCGACTACACTATTGGAT-3′ (#1) and KLF4, 5´-ATCGGTCATCAGCGTCAGCAA-3′ (#1); 5´-AAGTCATCTTGTGAGTGGATAA-3′ (#2).

### Real-time RT-PCR and RT-PCR

Total RNA was isolated using TRIzol (Invitrogen). One microgram of total RNA was used to synthesize cDNA using a PrimeScript™ RT reagent kit (Takara, RR047A) according to the manufacturer’s instructions. The sequences of the primers used are as follows: Actin F: 5´-GACCTGACTGACTACCTCATGAAGAT-3′ and R: 5´-GTCACACTTCATGATGGAGTTGAAGG-3′; OCT4 F: 5′- AGAACATGTGTAAGCTGCGG-3′ and R: 5´-GTTGCCTCTCACTCGGTTC-3′; Nanog F: 5´-GAAATACCTCAGCCTCCAGC-3′ and R: 5´-GCGTCACACCATTGCTATTC-3′; KLF4 F: 5´-ACCTACACAAAGAGTTCCCATC-3′, R: 5´-TGTGTTTACGGTAGTGCCTG-3′.

### Dual-luciferase reporter assay

The assay was performed as previously described [19].

### ChIP assay

The ChIP assay was performed as previously described [20].

### Immunoprecipitation and in vivo KLF4 ubiquitylation assay

Cells were transfected with the indicated plasmids using Lipofectamine 3000 (Invitrogen) reagent according to the manufacturer’s protocol. For immunoprecipitation assays, cells were lysed with NP40 lysis buffer (50 mM Tris-HCl, pH 8.0, 150 mM NaCl, 1% NP40, 0.5% deoxycholate) supplemented with protease-inhibitor cocktail (Biotool). Immunoprecipitations were performed using the KLF4 antibody and protein A/G agarose beads (Santa Cruz) at 4 °C. The immunocomplexes were then washed twice with 200 μL PBS. Both lysates and immunoprecipitates were examined using the indicated primary antibodies followed by detection with the corresponding secondary antibodies and Western Bright ECL chemiluminescent detection reagent (Advansta).

For in vivo deubiquitylation assays, HA-ubiquitin was transfected into LN229 cells, with or without ITGB4 knockdown, or overexpressed using Lipofectamine 3000. Twenty-four hours later, the cells were treated with 20 μM of the proteasome inhibitor MG132 (Calbiochem) for 8 h. Then, cells were lysed with NP40 lysis buffer and incubated with anti-KLF4 antibody for 3 h and protein A/G agarose beads (Santa Cruz) for a further 6 h at 4 °C. The beads were then washed three times with PBS buffer. The proteins were released from the beads by boiling in SDS-PAGE sample buffer and analysed by immunoblotting with anti-Ub monoclonal antibody.

### Protein half-life assay

For the KLF4 half-life assay, the LN229 cells with or without stably expressing ITGB4 or the indicated shRNAs, were treated with CHX (Sigma, 10 mg/mL) for the indicated durations before collection. The cell lysates were examined using the indicated primary antibodies followed by detection with the related secondary antibodies and Western Bright ECL chemiluminescent detection reagent (Advansta).

### Colony formation assay

LN229 and U251, with or without ITGB4 knockdown, were harvested and mixed by pipetting to become single-cell suspensions in complete culture media of a given concentration. Dilutions were made of the single-cell suspensions to 500 or 1000 cells in every well of 6-well plate. This was incubated at 37 °C with 5% CO_2_ for 2 weeks. The colonies were then stained with 0.04% crystal violet-2% ethanol in PBS. Photographs of the stained colonies were then taken.

### Cell migration assay

Cell migration assays were performed in 24-well transwell plates with 8-mm polyethylene terephalate membrane filters (Corning) separating the lower and upper culture chambers. In brief, LN229 or U251 cells were plated in the upper chamber at 1 × 10^4^ cells per well in serum-free DMEM medium. The bottom chamber contained DMEM medium with 10% FBS. Cells were allowed to migrate for 24 h in a humidified chamber at 37 °C with 5% CO_2_. After the incubation period, the filter was removed and non-migrant cells on the upper side of the filter were detached using a cotton swab. Filters were fixed with 4% formaldehyde for 15 min and cells located in the lower filter were stained with 0.1% crystal violet for 20 min and photographed.

### Tumour growth assay

Animal studies were conducted in accordance with the National Institute of Health Guide for the Care and Use of Laboratory Animals with the approval of the Animal Research Committee of Dalian Medical University. Male nude mice (4–6 weeks of age, 18–20 g) were obtained from the SPF Laboratory Animal Center of Dalian Medical University (Dalian, China) and were randomly divided into the indicated groups. The mice in each group (*n* = 6) were subcutaneously injected with the indicated cells. After 15 days, tumour size was measured every 5 days by Vernier callipers and converted to TV according to the following formula: TV (mm^3^) = (axb^2^)/2, where a and b are the largest and smallest diameters, respectively. All animals were killed 5 weeks after injection, and the transplanted tumours were removed, weighed, and fixed for further study.

### Tissue microarrays and immunohistochemistry

Glioma tissue microarrays were purchased from Alenabio and Shanghai Outdo Biotech Company. These contained a total of 112 glioma tissues and 8 normal tissues. Immunohistochemistry was performed as previously described [21]. The characteristics of the patients and their tumours were collected through the review of medical records and pathology reports. Informed consent with approval of the ethics committee of Taizhou Hospital of Zhejiang Province was obtained. All of the methods in this study were in accordance with the approved guidelines, and all of the experimental protocols were approved by the ethics committee of Taizhou Hospital of Zhejiang Province.

For immunohistochemistry, sections were subjected to antigen retrieval using microwave heating at 95 °C in citrate buffer (pH = 6.0). The indicated antibodies specific for KLF4 and ITGB4 were diluted according to the manufacturer’s instructions. The degrees of immunostaining were reviewed and scored by two independent observers. The proportion of the stained cells and the extent of the staining were used as the criteria for evaluation. For each case, at least 1000 tumour cells were analysed. For each sample, the proportion of KLF4 and ITGB4 expressing cells varied from 0 to 100%, and the intensity of staining varied from weak to strong. One score was given according to the percentage of positive cells as: < 5% of the cells: 1 point; 6–35% of the cells: 2 points; 36–70% of the cells: 3 points; > 70% of the cells: 4 points. Another score was given according to the intensity of staining as: negative staining: 1 point; weak staining (light yellow): 2 points; moderate staining (yellowish brown): 3 points; and strong staining (brown): 4 points. A final score was then calculated by multiplying the above two scores. If the final score was equal to or larger than four, the protein expression in the tumour was considered high; otherwise, the protein expression in the tumour was considered low.

We compared ITGB4 mRNA expression in normal tissues and in glioma tissue by searching the gene symbol “ITGB4 and fold change >1.5” in the Oncomine database. The data of 81 glioma tissues and 23 normal brain tissues were obtain from Sun brain (180) 204989_s_at; diffuse astrocytoma tissues (*n* = 7, Sun brain (180), 214292_at), anaplastic astrocytoma tissues (*n* = 19, Sun brain (180), 211905_s_at), and classic medulloblastoma (*n* = 46, Pomeroy brain (85) X53587_at).

### Statistics and data analyses

Statistical evaluations were performed using GraphPad Prism 5. Data are shown as mean ± SD. Multiple comparisons between treatment groups and controls were performed using Dunnett’s least significant difference (LSD) test. Statistical significance between groups was calculated using the LSD test in SPSS 17.0 software (IBM). The results of WB were analyzed using the Image J software. Values of *p* < 0.05 were considered statistically significant.

## Results

### ITGB4 expression is elevated in GSC-enriched populations

**Fig. 1:**
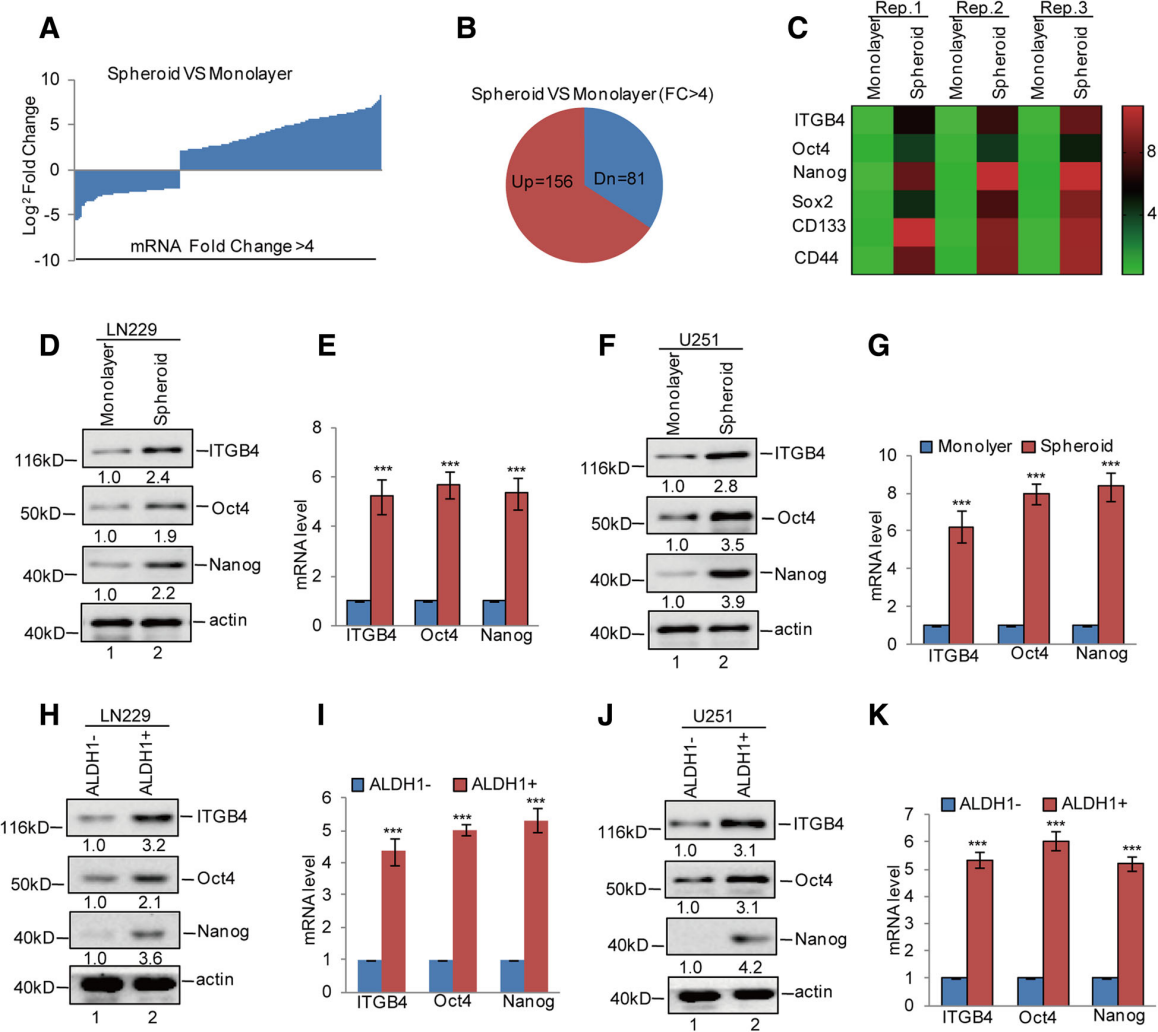
ITGB4 expression was increased in GSCs. **a**-**c** GSC-enriched populations were obtained from LN229 cells by sphere formation assay. The altered genes between spheroid and monolayer were detected by RNA sequencing analysis. The upregulated genes are listed. **d**-**g** The expression levels of ITGB4, Oct4, and Nanog were analysed by western blotting and q-RT-PCR assays. Data represent the mean ± SD of three independent experiments. *** *p* < 0.001 vs. control. **h**-**k** ALDH1 positive cells were obtained from LN229 and U251 cells by flow cytometry sorting. The expression levels of ITGB4, Oct4, and Nanog were analysed by western blotting and q-RT-PCR assays. Data represent the mean ± SD of three independent experiments. *** *p* < 0.001 vs. control

To identify the genes that are expressed differentially in GSCs, we first conducted a mammosphere formation assay to enrich GSCs from the glioma cell LN229 line, for subsequent mRNA sequencing analysis. Compared with the cells grown in monolayer cultures, we found 156 upregulated genes and 81 downregulated genes in GSCs (Fig. 1a and b). Among these genes, we found that ITGB4 expression was significantly increased in GSCs, which was confirmed by subsequent western blotting and q-RT-PCR assays. The upregulation of GSC markers Oct4, Nanog, Sox2, CD133, and CD44 were listed as the positive control (Fig. 1c-g). To further confirm this, we next enriched glioma stem cells from LN229 and U251 cells by ALDH1- positive sorting assay and found that ALDH1+ cells also exhibited much higher ITGB4 mRNA and protein expression levels than ALDH1− cells (Fig. 1h-k). Together, these data suggest that ITGB4 expression is elevated in GSC-enriched populations.

### ITGB4 is highly expressed in human glioma tissues and is positive correlated with glioma grades

**Fig. 2:**
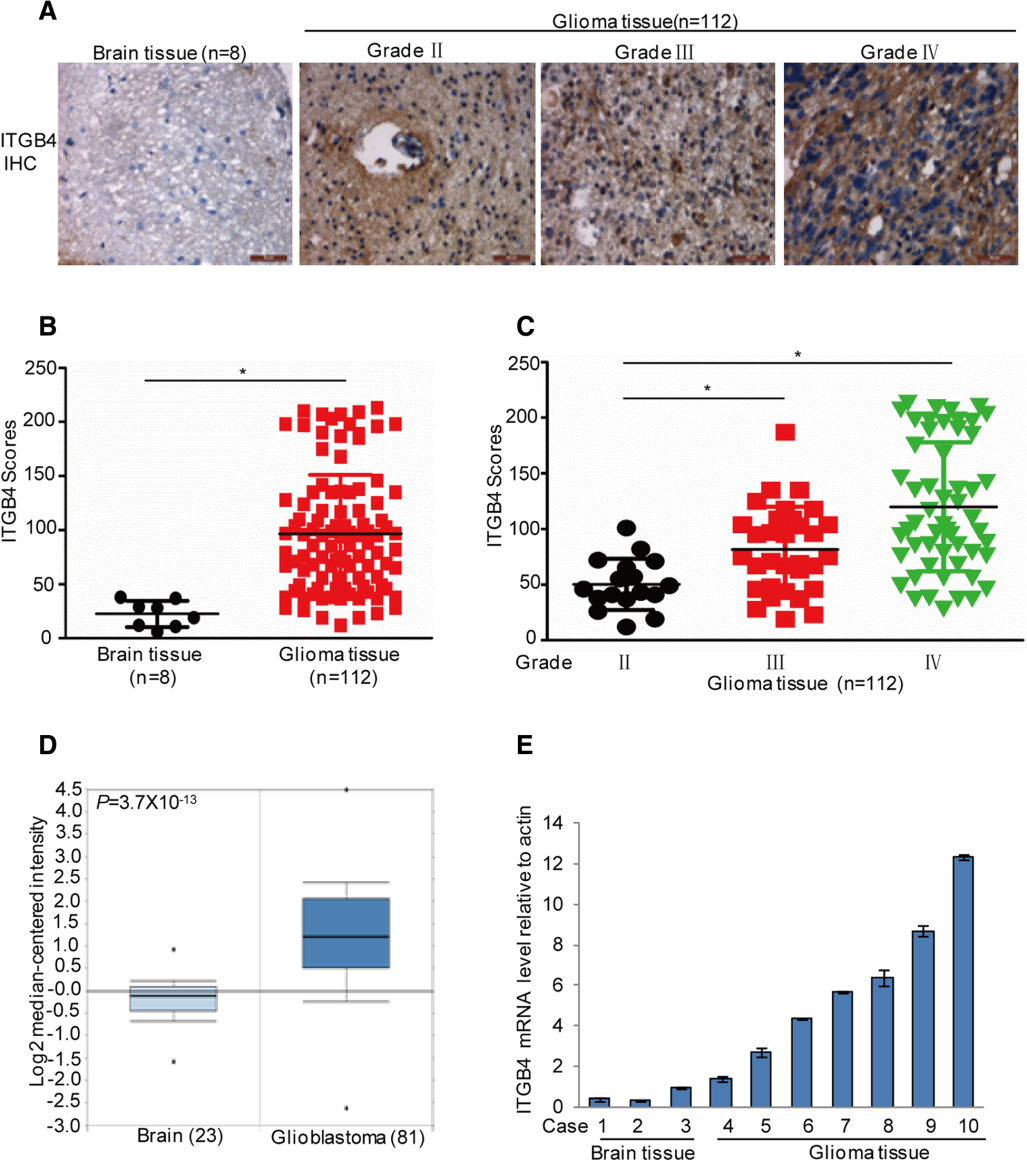
High expression of ITGB4 in glioma patients was associated with glioma grades. **a**-**c** Representative images from immunohistochemical staining of ITGB4. The expression levels of ITGB4 between normal tissues (*n* = 8) and glioma tissues (*n* = 112) were compared. The association between ITGB4 and the grades were analysed. * *p* < 0.05 vs. control. **d** ITGB4 mRNA expression in normal tissues (*n* = 23) and the glioblastoma tissues (*n* = 81) were analysed. The data were extracted from the Oncomine database. **e** The mRNA levels of ITGB4 between normal tissues (*n* = 3) and glioblastoma tissues were analysed by q-RT-PCR

To better understand the role of ITGB4 in the development of human glioma, we further examined ITGB4 expression in human glioma samples (*n* = 112; World Health Organization (WHO) grade II–IV) and nonneoplastic brain tissue samples (*n* = 8) by immunohistochemical (IHC) staining. The IHC staining indicated that ITGB4 expression levels were increased in human glioma samples relative to normal brain tissues (Fig. 2a-b). Interestingly, increased ITGB4 levels were found in high-grade gliomas and high ITGB4 expression was significantly correlated with increased tumour grade (Fig. 2c). Subsequently, the mRNA levels of ITGB4 were assessed in 81 glioma tissues and 23 normal brain tissues, using gene expression data obtained from the Oncomine database. Correspondingly, compared with normal brain tissues, ITGB4 mRNA levels were upregulated in glioblastoma tissues (Fig. 2d). Similar results were obtained in diffuse astrocytoma tissues (*n* = 7), anaplastic astrocytoma tissues (*n* = 19), and classic medulloblastoma (*n* = 46) (Additional file 1: Figure S1A-C). To further verify this, we examined the mRNA levels of ITGB4 in seven glioma tissues and three normal brain tissues from our own institution. Similarly, increased ITGB4 mRNA expression was observed in the glioma tissues (Fig. 2e).

### ITGB4 is an important mediator of the stem-like properties of GSCs

**Fig. 3:**
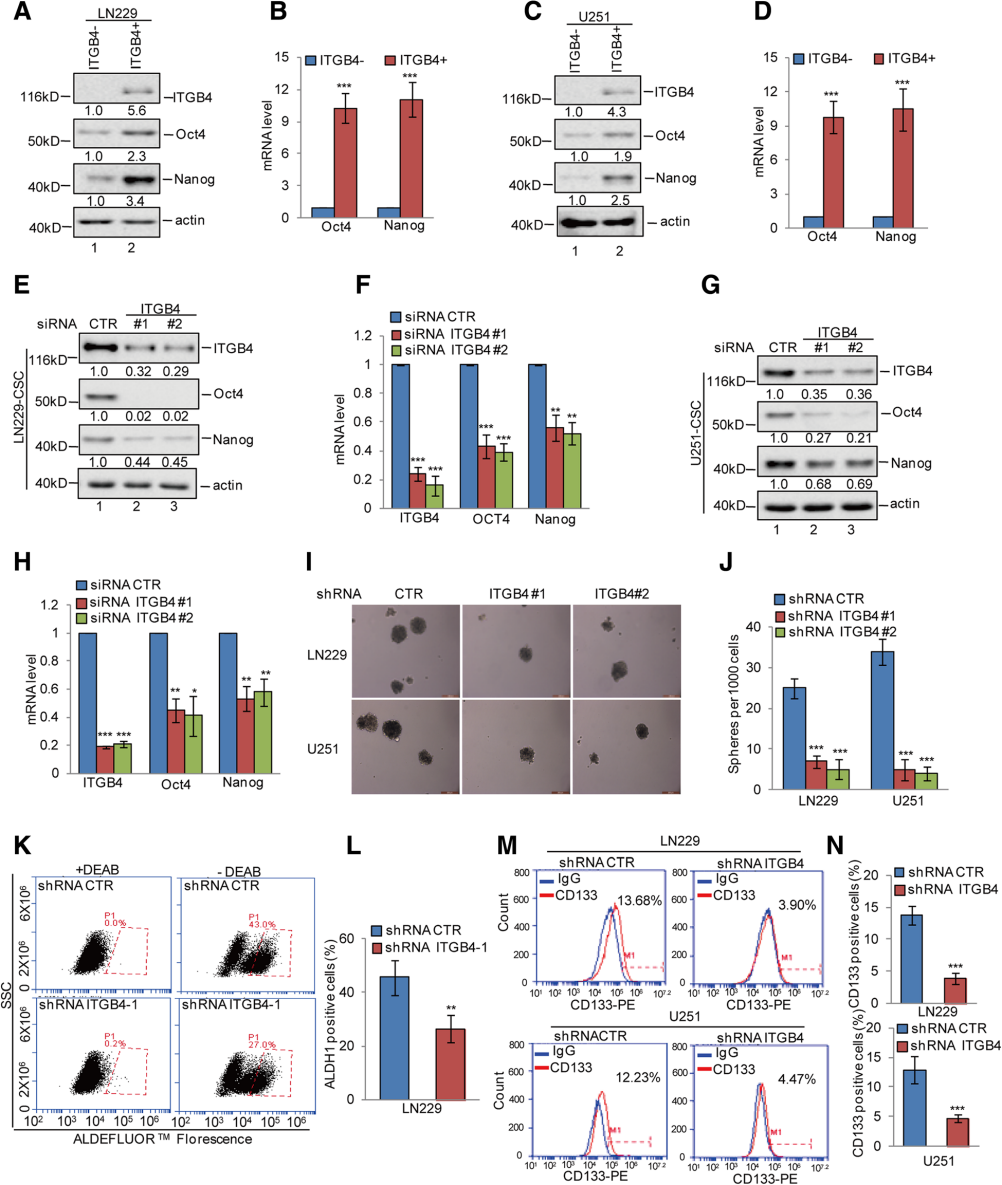
ITGB4 knockdown suppressed stem-like properties of glioma cells. **a**-**d** ITGB4+ and ITGB4- cells were isolated from LN229 and U251 cells by flow cytometry sorting. The expression levels of Oct4 and Nanog were analysed by western blotting and q-RT-PCR assays. Data represent the mean ± SD of three independent experiments. *** *p* < 0.001 vs. control. **e**-**h** GSCs were enriched from LN229 and U251 cells by sphere formation assay. We then knocked down ITGB4 expression using siRNA in the GSCs. The expression levels of ITGB4, Oct4, and Nanog were analysed by western blotting and q-RT-PCR assays. Data represent the mean ± SD of three independent experiments. ** *p* < 0.01, *** *p* < 0.001 vs. control. **i**-**n** ITGB4 was knocked down in LN229 and U251 cells. The mammosphere-forming abilities, ALDH1-positive populations, and CD133-positive populations were analysed. Data represent the mean ± SD of three independent experiments. ** *p* < 0.01, *** *p* < 0.001 vs. control

To assess the contribution of ITGB4 in promoting glioma stem-like properties, we first obtained ITGB4-positive cells from the LN229 and U251 cells by fluorescence-activated cell sorting (FACS). Compared with the ITGB4-negative cells, ITGB4 positive cells exhibited much higher Oct4 and Nanog expression (Fig. 3a-d). To further investigate the role of ITGB4 in GSCs, we enriched cancer stem cells from LN229 and U251 cells by mammosphere formation assay and then knocked down ITGB4 expression in these cells using siRNA. Compared with the control group, ITGB4 knockdown remarkably decreased the expression of the Oct4 and Nanog stemness markers (Fig. 3e-h).

Next, we stably knocked down ITGB4 expression using lentivirus expressing short-hairpin RNA (shRNA) in LN229 and U251 cells. As shown in Additional file 2: Figure S2A-B, stable cell lines expressing these shRNAs showed significantly reduced ITGB4 levels. We then performed mammosphere formation, ALDH1-positive, and CD133-positive sorting assays to investigate the role of ITGB4 in promoting self-renewal abilities, a key characteristic of GSCs. The sphere formation efficiency was dramatically reduced upon ITGB4 depletion, as indicated by a decrease in spheroid numbers (Fig. 3i-j). Additionally, the ALDH1+ and CD133+ population was markedly decreased following the depletion of ITGB4 (Fig. 3k-n). Together, these data implied that ITGB4 is necessary for promoting cancer stem-like properties.

### ITGB4 knockdown suppressed glioma cell migration and proliferation in vitro and in vivo

**Fig. 4:**
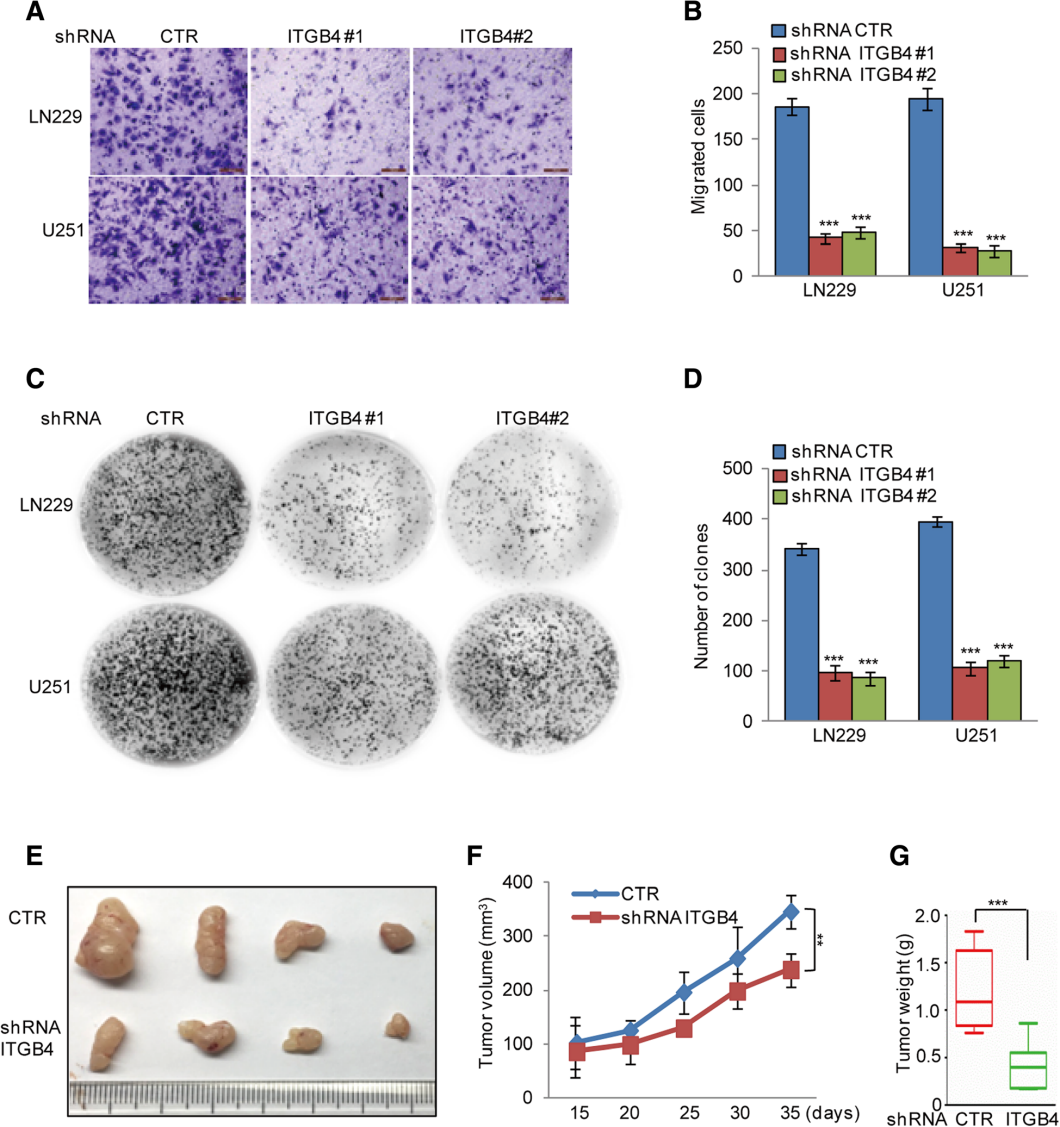
ITGB4 knockdown suppressed glioma cell migration and proliferation. **a**-**d** ITGB4 was knocked down in LN229 and U251 cells. The cell migration and proliferation were analysed by transwell and colony formation assays. Data represent the mean ± SD of three independent experiments. *** *p* < 0.001 vs. control. **e**-**g** LN229 cells with or without ITGB4 knockdown were subcutaneously injected into nude mice (*n* = 6 in each group) for tumour formation. Representative bright-field imaging of the tumours in the mice implanted the indicated cells. After 5 weeks, mice receiving transplants of the indicated cells were sacrificed. The tumour volume and weight were calculated. ** *p* < 0.01, *** *p* < 0.001 vs. control

Given that ITGB4 suppression diminished GSC properties in vitro, we then examined whether ITGB4 knockdown could affect glioma cell migration and proliferation in vitro and in vivo. To this end, we used the LN229 and U251 cell lines and stably knocked down ITGB4 with two independent shRNAs. In the subsequent transwell assay, the migratory capabilities of LN229 and U251 cells depleted of ITGB4 were apparently decreased compared to control cells (Fig. 4a-b).

Furthermore, the numbers of colonies formed by LN229 and U251 cells with ITGB4 knockdown were also remarkably reduced compared to control cells (Fig. 4c-d). To examine the involvement of ITGB4 expression in glioma tumorigenesis in vivo, we implanted LN229 cells, stably expressing control shRNA or shRNA targeting ITGB4, into nude mice. As illustrated in Fig. 4e-g, the size and weight of xenograft tumours were significantly reduced by the ITGB4 knockdown.

### KLF4 upregulates ITGB4 expression in glioma cells

**Fig. 5:**
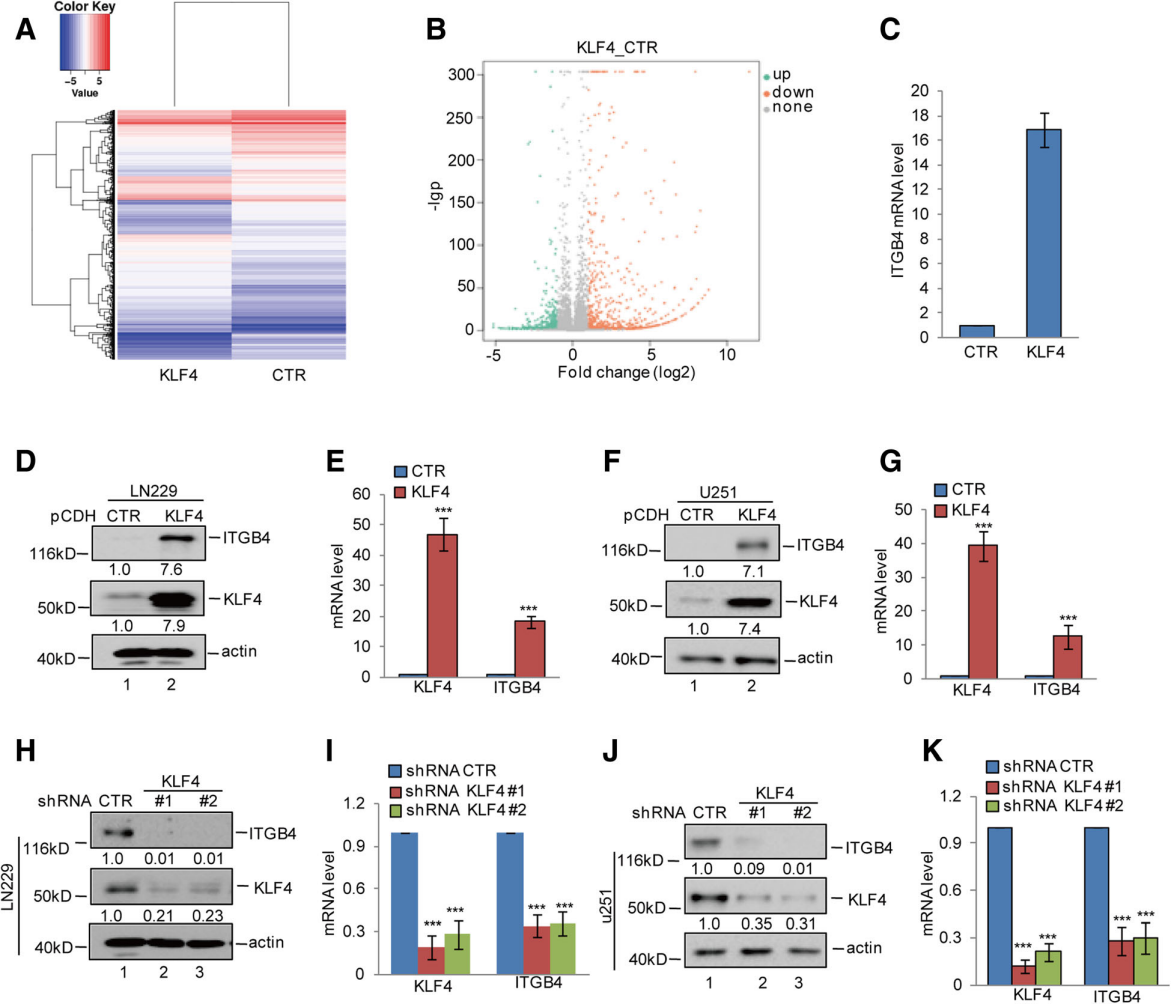
KLF4 upregulated ITGB4 expression. **a**-**c** KLF4 was overexpressed in LN229 cells. Gene expression profiles were obtained by RNA sequencing analysis. The mRNA levels of ITGB4 are listed. **d**-**g** KLF4 was overexpressed in LN229 and U251 cells. The expression levels of ITGB4 and KLF4 were detected by western blotting and q-RT-PCR assays. Data represent the mean ± SD of three independent experiments. *** *p* < 0.001 vs. control. **h**-**k** KLF4 was knocked down in LN229 and U251 cells. The expression levels of ITGB4 and KLF4 were detected by western blotting and q-RT-PCR assays. Data represent the mean ± SD of three independent experiments. *** *p* < 0.001 vs. control

KLF4/GKLF is a member of the KLF-like factor subfamily of zinc finger proteins [22]. Our recent studies have indicated that KLF4 upregulates MGLL and BIK in HCC and prostate cancer cells [23–25]. To screen the KLF4-regulated genes in glioma, we overexpressed KLF4 in LN229 cells for subsequent mRNA sequencing analysis. Interestingly, among the upregulated genes, we found that ITGB4 was significantly elevated in KLF4 overexpressing cells (Fig. 5a-c).

To further confirm this, we first detected the mRNA and protein levels of ITGB4 in KLF4 overexpressing LN229 and U251 cells. Compared with the control cells, overexpression of KLF4 significantly increased ITGB4 expression (Fig. 5d-g). Conversely, inhibition of KLF4 dramatically decreased ITGB4 expression (Fig. 5h-k).

### KLF4 directly binds to the promoter of ITGB4

**Fig. 6:**
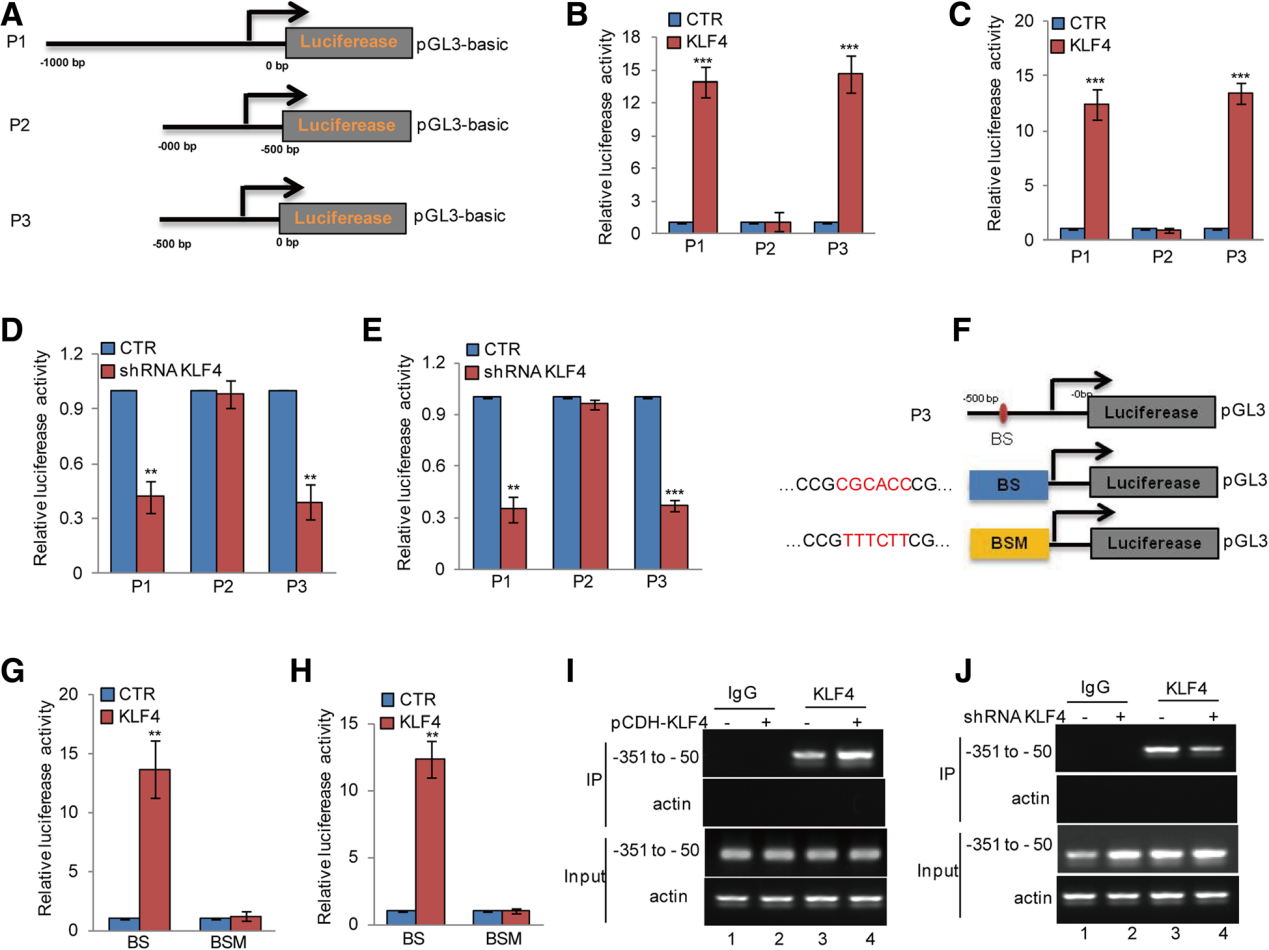
KLF4 binds to the promoter of ITGB4. **a** Schematic illustration of pGL3-based reporter constructs were used in luciferase assays to examine the transcriptional activity of ITGB4. **b**-**c** Parts of the promoter of ITGB4, named P1, P2, and P3, were individually transfected into LN229 and U251 cells with or without KLF4 overexpression. Luciferase activity was measured. Data represent the mean ± SD of three independent experiments. *** *p* < 0.001 vs. control. **d**-**e** Parts of the promoter of ITGB4, named P1, P2, and P3, were individually transfected into LN229 and U251 cells with or without KLF4 knockdown. Luciferase activity was measured. Data represent the mean ± SD of three independent experiments. ** *p* < 0.01, *** *p* < 0.001 vs. control. **f** The potential KLF4 binding site was inspected by JASPAR. Schematic illustration of KLF4 wild type binding site (BS) and the matching mutant (BSM) that were used in luciferase assays. **g**-**h** The wild type promoter (BS) or the matching mutant (BSM) were individually transfected into LN229 and U251 cells with or without KLF4 overexpression. Luciferase activity was measured. Data represent the mean ± SD of three independent experiments. ** p < 0.01 vs. control. **i**-**j** ChIP analysis showing the binding of KLF4 to the promoter of ITGB4 in LN229 cells with or without KLF4 overexpression or knockdown. An isotype-matched IgG was used as a negative control

To identify the KLF4 binding regions on the ITGB4 promoter, we first cloned the upstream sequence of ITGB4 and different truncations of it. We then inserted them into pGL3-based luciferase reporter plasmids, named P1–P3 (Fig. 6a). We transfected them into LN229 and U251 cells with or without KLF4 overexpression. Compared with control cells, the luciferase activities of P1 and P3 were augmented in KLF4 overexpressing cells; however, this increase was abolished when P2 was transfected (Fig. 6b-c). To further verify this, the truncations were transfected into LN229 and U251 cells with or without KLF4 knockdown. We found that KLF4 depletion led to a decrease in luciferase activity from P1 and P3. However, this decrease disappeared when P2 was transfected in KLF4 knockdown cells (Fig. 6d-e). Taken together, these results indicate that the region from − 500 to 0 bp (P3) is a key region for the promotion of ITGB4 by KLF4.

Previous reports have indicated that KLF4 is a zinc finger-type transcription factor that usually binds to the GC rich elements of promoters [26]. To identify potential KLF4 binding sites, we inspected the sequence of the ITGB4 promoter by JASPAR software and found a putative KLF4 binding site on the ITGB4 promoter. To verify that this potential KLF4-binding site was indeed responsive to KLF4, two pGL3-based luciferase reporter plasmids named BS and BSM were established (Fig. 6f). These plasmids were individually transfected into LN229 and U251 cells with or without KLF4 knockdown. As shown in Fig. 6g and h, the luciferase activity of BS but not BSM was significantly increased in KLF4 overexpressing cells. In addition, subsequent chromatin immunoprecipitation (ChIP) assays showed that the chromatin fragments corresponding to the putative KLF4 binding sites were specifically present in the anti-KLF4 immunoprecipitates from LN229 cells. This bond was increased when KLF4 was overexpressed, whereas, the bond was decreased when KLF4 was knocked down (Fig. 6i-j).

### ITGB4 interacts with KLF4 and enhances its stability

**Fig. 7:**
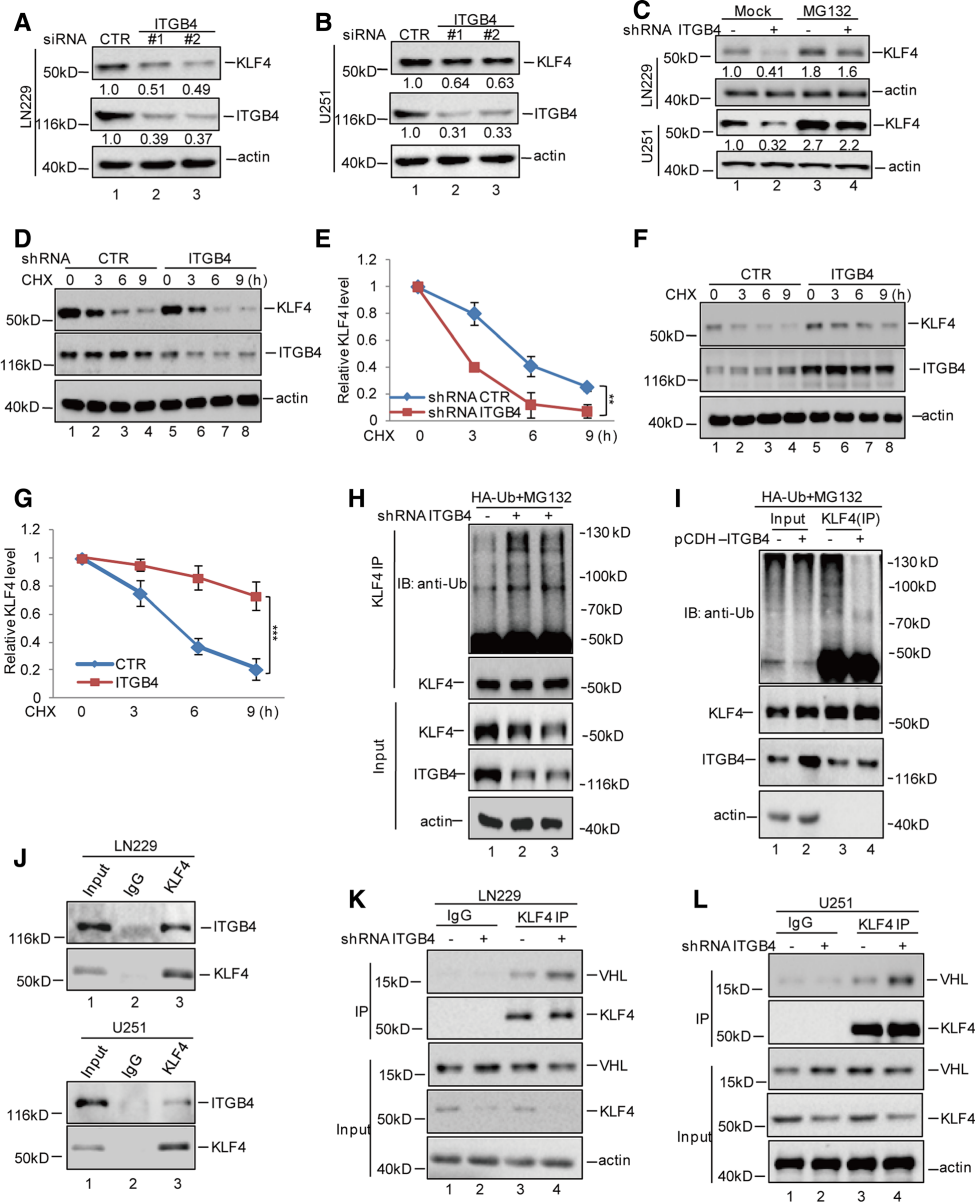
ITGB4 enhanced KLF4 stability. **a**-**b** ITGB4 was knocked down in LN229 and U251 cells. The protein levels of ITGB4 and KLF4 were analysed by western blotting assay. **c** LN229 and U251 cells with or without ITGB4 knockdown were treated with MG132 or not. The protein levels of ITGB4 and KLF4 were analysed by western blotting assay. **d**-**g** LN229 cells with or without ITGB4 knockdown or overexpression were treated with CHX (10 mg/ml) for the indicated times. The half-life of KLF4 was measured. Data represent the mean ± SD of three independent experiments. ** *p* < 0.01, *** *p* < 0.001 vs. control. **h**-**i** LN229 cells with or without ITGB4 knockdown or overexpression, were transfected with the indicated constructs and the cells then were treated with MG132 for 8 h before collection. The whole-cell lysate was subjected to immunoprecipitation with anti-KLF4 antibodies and western blotted with anti-Ub antibodies to detect ubiquitylated KLF4. **j** LN229 and U251 cell lysates were subjected to immunoprecipitation with control IgG or anti-KLF4 antibodies. The immunoprecipitates were then detected using the indicated antibodies. **k**-**l** ITGB4 was knocked down or overexpressed in LN229 cells. The cell lysates were subject to immunoprecipitation with control IgG or anti-KLF4 antibodies. The immunoprecipitates were then detected using the indicated antibodies

In addition to KLF4 being identified as a potential regulator of ITGB4, we also found that a positive feedback loop existed between KLF4 and ITGB4. As shown in Fig. 7a and b, we found that the knockdown of ITGB4 using siRNA led to KLF4 downregulation and that the decrease in KLF4 was restored by the proteasome inhibitor MG132. This indicated that ITGB4 affected KLF4 expression in a proteasome-dependent manner (Fig. 7c). To further validate this finding, we treated the indicated cells with the protein synthesis inhibitor cycloheximide (CHX). Notably, the depletion of ITGB4 led to a prominent decrease in the stability of endogenous KLF4 protein (Fig. 7d-e). Whereas, overexpression of ITGB4 enhanced the stability of KLF4 in LN229 cells (Fig. 7f-g). Additionally, we also found that knockdown of ITGB4 increased the ubiquitylation of KLF4 in glioma cells (Fig. 7h), while overexpression of ITGB4 decreased the ubiquitylation of KLF4 (Fig. 7i).

To uncover the molecular mechanism by which ITGB4 enhances KLF4 stability, we first wanted to investigate whether ITGB4 could interact with KLF4. The binding assays suggested that ITGB4 could interact with KLF4 in glioma cells and they were co-localization in the cells (Fig. 7 and Additional file 3: Figure S3). Subsequently, we also found that the binding of KLF4 to VHL, an E3 ligase of KLF4, was increased in ITGB4 knockdown cells (Fig. 7k). Similar to the knockdown assay, the interaction between KLF4 and VHL was decreased in ITGB4 overexpressing cells (Fig. 7l). Taken together, our data suggest that ITGB4 interacts with KLF4 and prevents its degradation.

### Reciprocal regulation between KLF4 and ITGB4 plays an essential role in glioma stem cell self-renewal and tumourigenesis

**Fig. 8:**
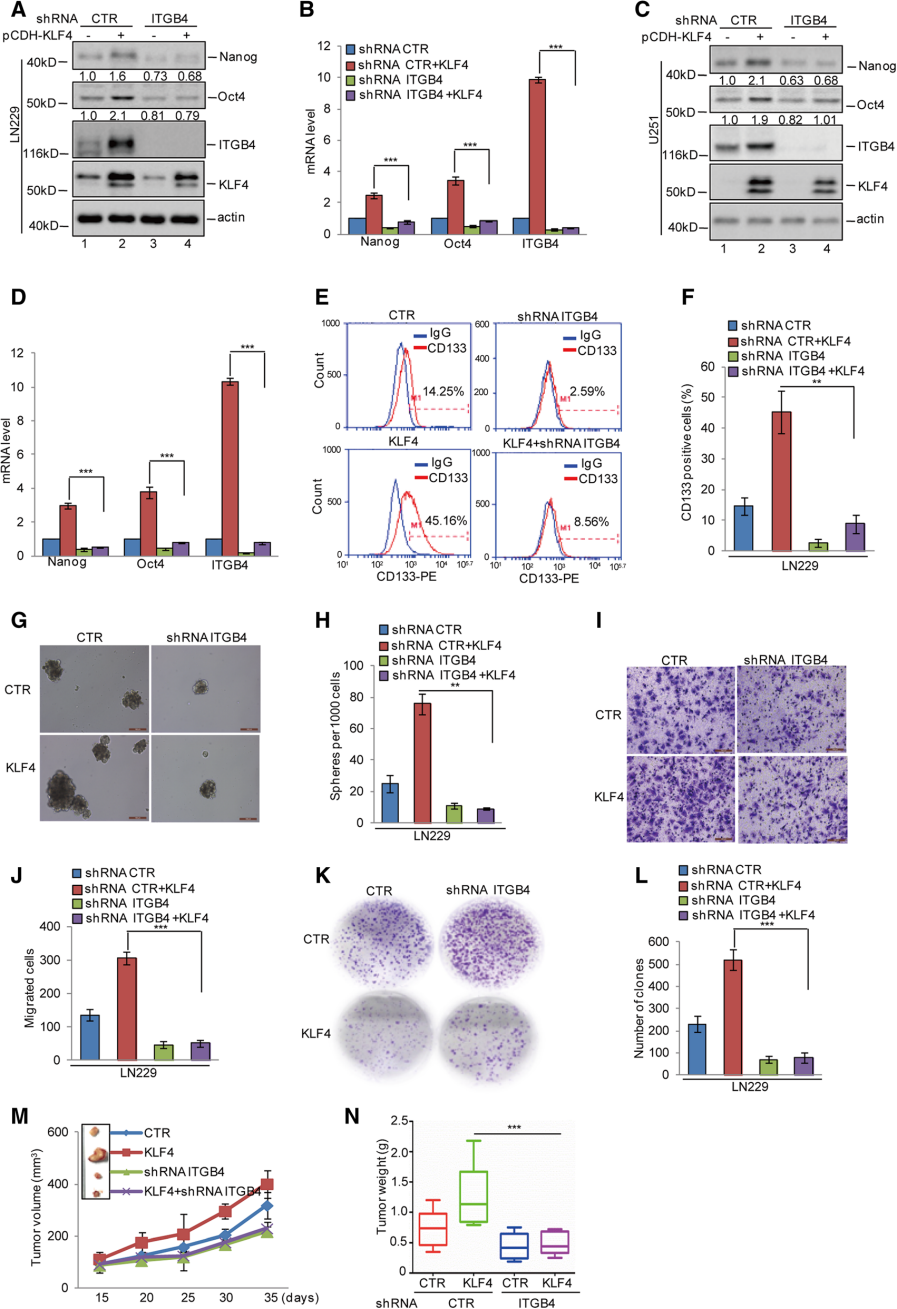
KLF4 and ITGB4 played an essential role in glioma tumorigenesis. **a**-**d** ITGB4 was knocked down in LN229 and U251 cells with or without overexpressing KLF4. The expression levels of ITGB4, Oct4, Nanog, and KLF4 were analysed by western blotting and q-RT-PCR assays. Data represent the mean ± SD of three independent experiments. *** *p* < 0.001 vs. control. **e**-**h** The CD133-positive populations and mammosphere-forming abilities were analysed. Data represent the mean ± SD of three independent experiments. ** *p* < 0.01 vs. control. **i-l** Cell migration and proliferation were analysed by transwell and colony formation assays. Data represent the mean ± SD of three independent experiments. *** *p* < 0.001 vs. control. **m**-**n** The cells were subcutaneously injected into nude mice (*n* = 6 in each group) for tumour formation. Representative bright-field imaging of the tumours in the mice implanted with the indicated cells. After 5 weeks, mice receiving transplants of the indicated cells were sacrificed. The tumour volume and weight were calculated. ** *p* < 0.01, *** *p* < 0.001 vs. control

Having identified that the feedback loop existed between KLF4 and ITGB4, we next asked whether reciprocal regulation between KLF4 and ITGB4 facilitated GSC self-renewal, proliferation, and migration. We first knocked down ITGB4 in LN229 and U251 cells with or without KLF4 overexpression. The expression levels of Oct4 and Nanog were analysed by western blotting and q-RT-PCR. As shown in Fig. 8a-d, KLF4 overexpression elevated the expression of Oct4 and Nanog. However, the upregulation of Oct4 and Nanog disappeared when ITGB4 was knocked down. After this, to assess the contribution of KLF4 in promoting GSC properties through regulation of ITGB4, CD133-positive sorting assays, mammosphere formation, transwell assays, and plate colony formation were performed. As shown in Fig. 8e-l, the elevations of the CD133+ population, sphere formation efficiency, migration ability, and colony numbers induced by KLF4 were reversed when ITGB4 was knocked down. To further confirm this, we stably overexpressed KLF4 in LN229 cells with or without ITGB4 knockdown, then implanted these cells into nude mice. As shown in Fig. 8m and n, we found that KLF4 overexpression promoted glioma growth as indicated by the increase in the size and weight of xenograft tumours. However, this increase was abolished when ITGB4 was depleted.

### KLF4 is closely associated with glioma grades and ITGB4 expression in glioma tissues

**Fig. 9:**
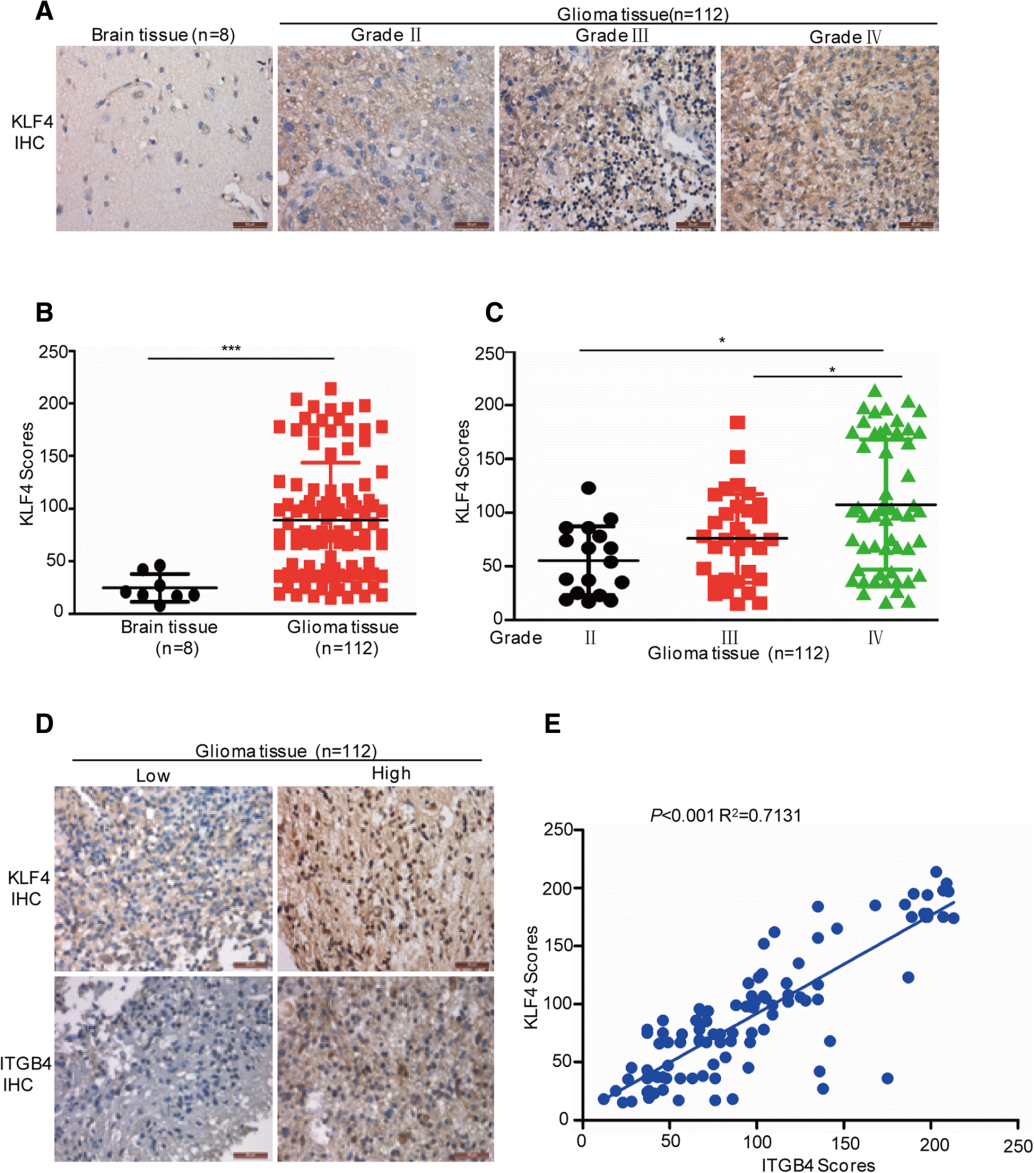
KLF4 was positively associated with ITGB4 expression in glioma tissues. **a**-**c** Representative images from the immunohistochemical staining of KLF4. The expression levels of KLF4 between normal tissues (*n* = 8) and glioma tissues (*n* = 112) were compared. The association between KLF4 and the grades were analysed. * *p* < 0.05, *** *p* < 0.001 vs. control. **d**-**e** Representative images from the immunohistochemical staining of KLF4 and ITGB4 in glioma tissues (*n* = 112). The association between KLF4 and ITGB4 was analysed (*p* < 0.001, R^2^ = 0.7131)

To evaluate the clinical relevance of the KLF4-ITGB4 axis, we first examined the expression of KLF4 in human glioma samples (*n* = 112; World Health Organization (WHO) grade II–IV) and nonneoplastic brain tissue samples (*n* = 8) by immunohistochemical (IHC) staining. These results showed that KLF4 displayed higher expression levels at varying degrees, compared with nonneoplastic brain tissues. High expression of KLF4 was significantly correlated with increased tumour grade (Fig. 9a-c). In addition, we further analysed the correlation between KLF4 and ITGB4 in human glioma tissues (n = 112). As shown in Fig. 9d and e, we found that KLF4 was closely associated with ITGB4 expression (*p* < 0.001, R^2^ = 0.7131).

## Discussion

Most patients with glioma tend to show recurrence and poor prognosis, which are believed to be closely related to the existence of GSCs. Therefore, it is necessary to understand how GSCs maintain their stem-like properties and find GSC-related therapeutic targets to improve glioma prognosis [27–31]. In this study, our findings showed that ITGB4 expression was increased in GSCs and glioma tissues. Elevated ITGB4 levels were correlated with glioma grades. Subsequently, we found that ITGB4 knockdown decreased the self-renewal abilities of GSCs and suppressed glioma cell migration and proliferation in vitro and in vivo. Further mechanistic studies revealed that KLF4, an important transcription factor, directly binds to the promoter of ITGB4, facilitating its transcription and contributing to increased ITGB4 expression in glioma. Interestingly, when ITGB4 levels were increased, it was able to interact with KLF4 and thus decrease its binding to the E3 ligase VHL, leading to its accumulation in glioma.

There is increasing evidence to indicate that the overexpression of ITGB4 is correlated with an aggressive phenotype and poor prognosis in breast cancer, lung cancer, pancreatic cancer, cervical cancer, and gastric cancer [17]. Similarly, we found that the mRNA and protein levels of ITGB4 were increased in human glioma and GSCs. Increased ITGB4 levels played an oncogenic role in glioma and were correlated with the glioma grades. Inhibition of ITGB4 expression in glioma cells reduced the self-renewal abilities of GSCs and suppressed glioma cell migration and proliferation in vitro and in vivo.

Recently, Transmembrane protein 268 (TMEM268) was reported to increase ITGB4 expression via enhancing its stability in gastric cancer cells [32, 33]. TAp73 was indicated to transcriptionally upregulate ITGB4 expression [34]. Here, we found that KLF4 directly binds to the promoter of ITGB4, facilitating its transcription and contributed to ITGB4 increase in glioma.

KLF4 is a member of the KLF-like factor subfamily of zinc finger proteins [22]. Dysregulation of KLF4 has been observed in a number of human cancers, including gastrointestinal, pancreas, bladder, and lung cancer. Ectopic expression of KLF4 has been reported to suppress cell proliferation, induce apoptosis, and promote cell-cycle arrest, indicating that KLF4 has a tumour suppressor function in a variety of malignancies and that its downregulation may play an essential role in tumourigenesis [35–40]. For example, KLF4 was reported to suppress cell migration and invasion in esophageal cancer [41, 42]. In gastric cancer, KLF4 inhibited cell proliferation and metastasis via downregulating β-catenin expression [43–45]. Recently, KLF4 was reported to transcriptionally repress cavelion-1 expression and thereby inhibit metastasis of pancreatic cancer [46]. However, in squamous cell carcinoma, breast cancer, osteosarcoma, and glioma, KLF4 was shown to promote cell growth and cellular dedifferentiation, and inhibit cell apoptosis [47–49]. Consistently, our data indicated that KLF4 promoted gliomagenesis via upregulating ITGB4 expression.

Interestingly, we also found that a positive feedback loop existed between ITGB4 and KLF4. ITGB4 was able to interact with KLF4 and enhanced its stability. Previous studies have indicated that the E3 ligase VHL and FOXB33 promoted KLF4 degradation in breast cancer [50, 51]. Our data suggests that ITGB4 binds to KLF4 and suppresses its interaction with VHL.

## Conclusions

Taken together, our data suggests the existence of a positive feedback loop between KLF4 and ITGB4 that promotes GSC self-renewal and gliomagenesis, and also implicates ITGB4 as a valuable therapeutic target for glioma.

## Additional files

Additional file 1: **Figure S1.** (A) ITGB4 mRNA expression in brain tissues (*n* = 23) and diffuse astrocytoma tissues (*n* = 7) were analysed. (B) ITGB4 mRNA expression in brain tissues (*n* = 23) and anaplastic astrocytoma tissues (*n* = 19) were analysed. (C) ITGB4 mRNA expression in cerebellum (*n* = 4) and classic medulloblastoma (*n* = 23) were analysed. These data were extracted from the Oncomine database. (TIF 108 kb)

Additional file 2: **Figure S2.** (A-D) ITGB4 was knocked down in LN229 and U251 cells using shRNA. The expression levels of ITGB4 were analysed by western blotting and q-RT-PCR. (TIF 268 kb)

Additional file 3: **Figure S3.** nuclear and cytosol proteins were isolated from LN229 cells. The expression levels of ITGB4 and KLF4 were detected by western blotting. PARP was used as nuclear marker and GAPDH was used as cytoplasm marker. (TIF 39 kb)

## Abbreviations

GSCs: Glioma stem cells
ITGB4: Integrin β4
KLF4: Kruppel-like Factor 4
Oct-4: Octamer-binding transcription factor 4
VHL: Von Hippel-Lindau
