# Correction: Reciprocal regulation of integrin β4 and KLF4 promotes gliomagenesis through maintaining cancer stem cell traits

**DOI:** 10.1186/s13046-023-02907-7

**Published:** 2023-11-29

**Authors:** Binbin Ma, Li Zhang, Yujie Zou, Ruiping He, Qiong Wu, Chuanchun Han, Bo Zhang

**Affiliations:** 1 Department of Neurosurgery, Second Affiliated Hospital, Institute of Cancer Stem Cell, Dalian Medical University, Dalian, 116027 China; 2 Laboratory of Pathogenic Biology, College of Basic Medical Science, Dalian Medical University, Dalian, 116027 China; 3 Nursing Department, First Affiliated Hospital, Dalian Medical University, Dalian, 116011 China; 4 Department of Neurology of Dalian Municipal Central Hospital Affiliated, Dalian Medical University, Dalian, 116033 China

**Correction:** ***J Exp Clin Cancer Res*** **38, 23 (2019)**

**https://doi.org/10.1186/s13046-019-1034-1**

Following the publication of the original article [1], errors were found in the images of Figs. 3, 4, 7, and 8, specifically:

- Fig. 3G – incorrect band of ITGB4 was used
- Fig. 4A – incorrect image of shRNA ITGB4#2 of U251 cell was used
- Fig. 7F –The direction of actin was reversed
- Fig. 8M – *p*-Value was lost

The correct figures are given below:

**Fig. 3:**
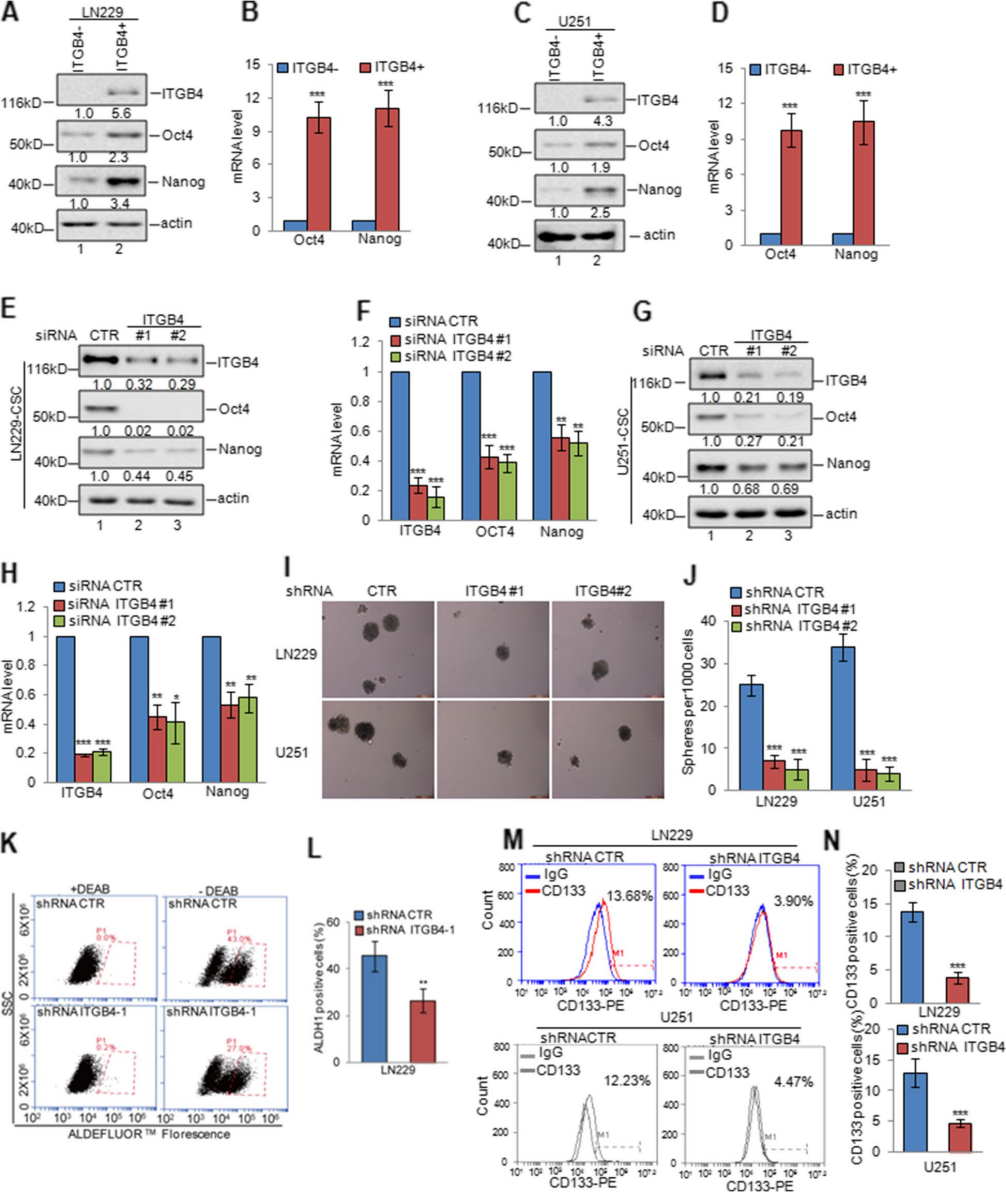
ITGB4 knockdown suppressed stem-like properties of glioma cells. **a-d** ITGB4 + and ITGB4- cells were isolated from LN229 and U251 cells by flow cytometry sorting. The expression levels of Oct4 and Nanog were analysed by western blotting and q-RT-PCR assays. Data represent the mean ± SD of three independent experiments. *** *p* < 0.001 vs. control. **e–h** GSCs were enriched from LN229 and U251 cells by sphere formation assay. We then knocked down ITGB4 expression using siRNA in the GSCs. The expression levels of ITGB4, Oct4, and Nanog were analysed by western blotting and q-RT-PCR assays. Data represent the mean ± SD of three independent experiments. ** *p* < 0.01, *** *p* < 0.001 vs. control. **i-n** ITGB4 was knocked down in LN229 and U251 cells. The mammosphere-forming abilities, ALDH1-positive populations, and CD133-positive populations were analysed. Data represent the mean ± SD of three independent experiments. ** *p* < 0.01, *** *p* < 0.001 vs. control

**Fig. 4:**
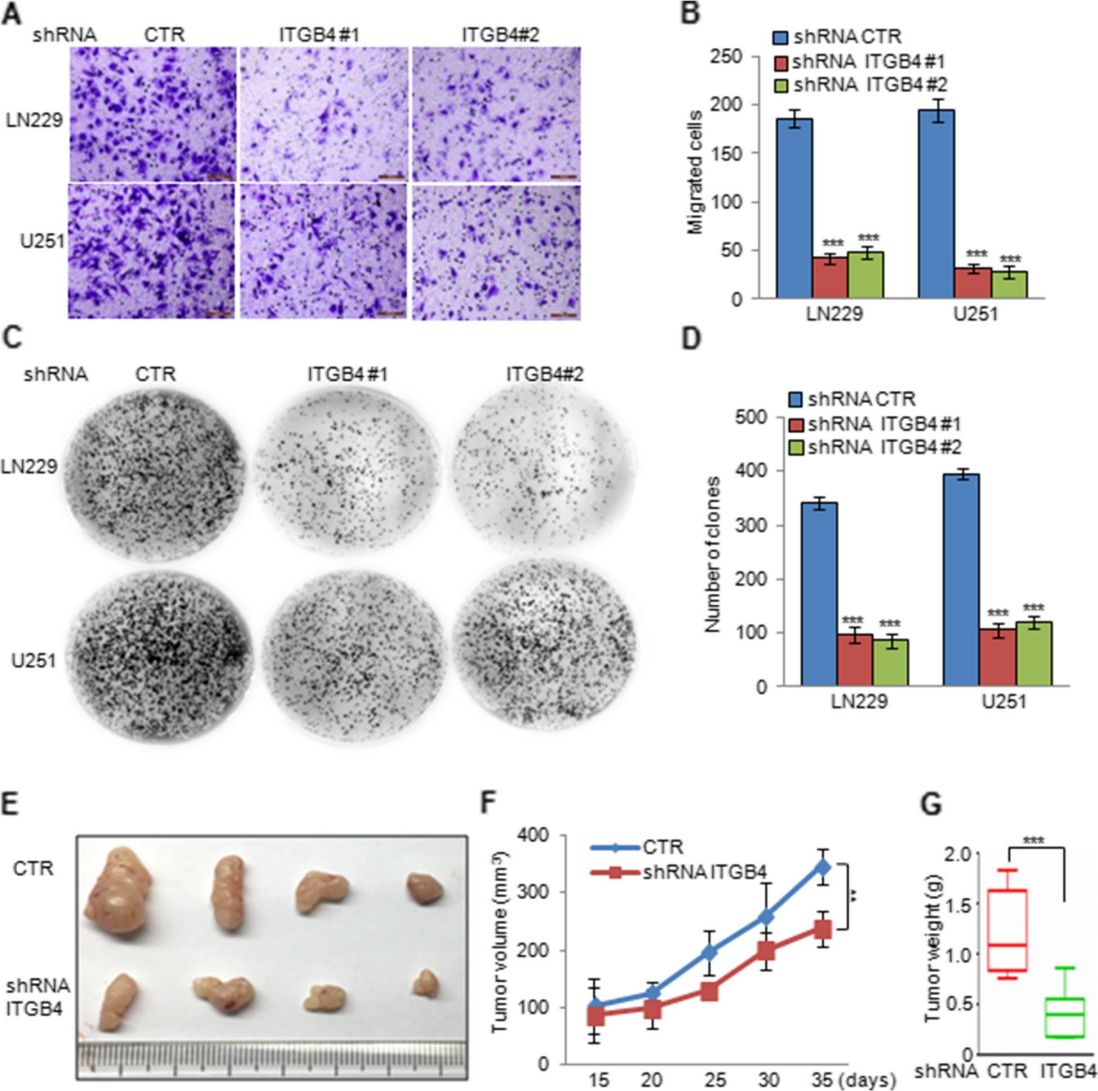
ITGB4 knockdown suppressed glioma cell migration and proliferation. **a-d** ITGB4 was knocked down in LN229 and U251 cells. The cell migration and proliferation were analysed by transwell and colony formation assays. Data represent the mean ± SD of three independent experiments. *** *p* < 0.001 vs. control. **e–g** LN229 cells with or without ITGB4 knockdown were subcutaneously injected into nude mice (n = 6 in each group) for tumour formation. Representative bright-field imaging of the tumours in the mice implanted the indicated cells. After 5 weeks, mice receiving transplants of the indicated cells were sacrificed. The tumour volume and weight were calculated. ** *p* < 0.01, *** *p* < 0.001 vs. control

**Fig. 7:**
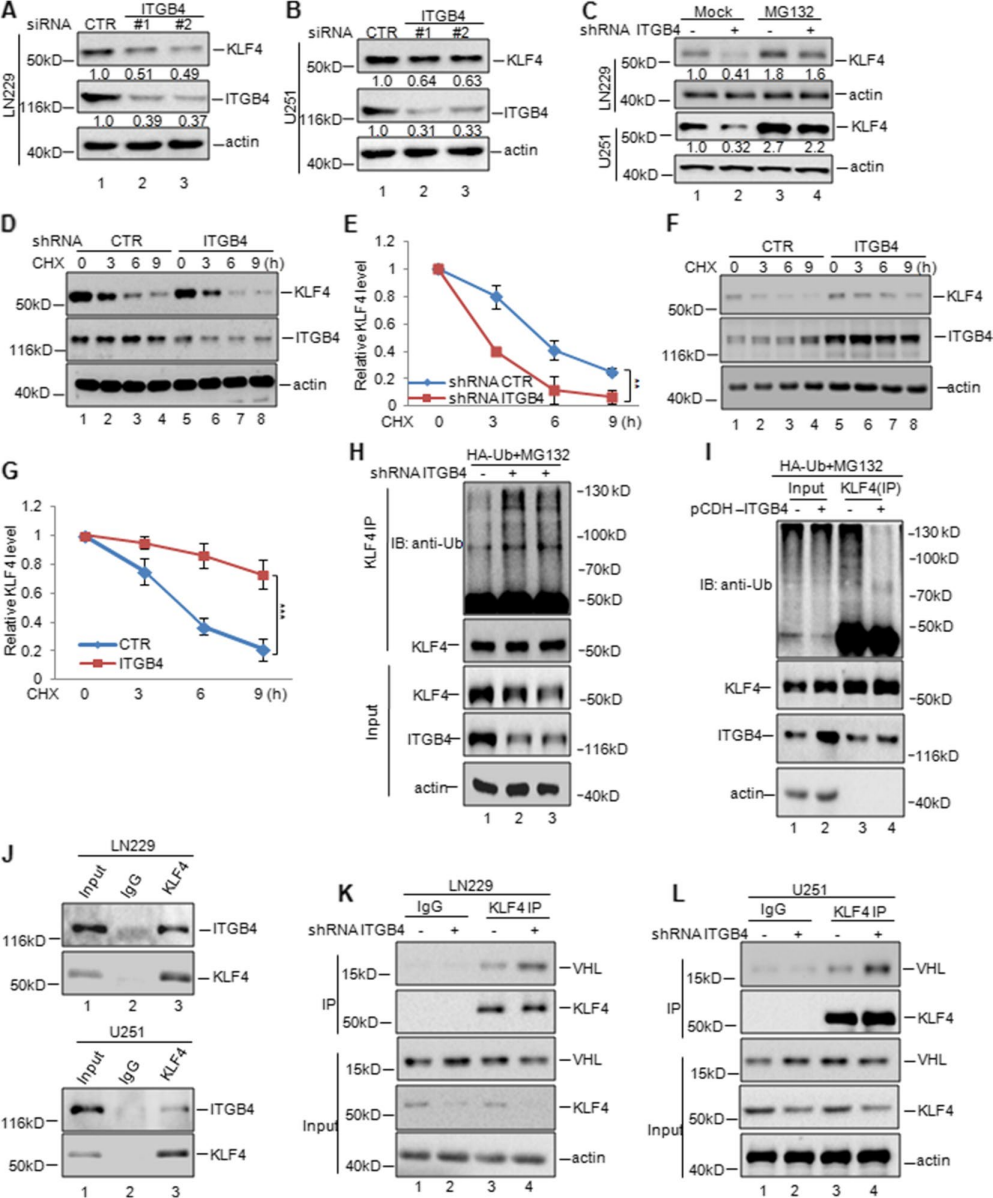
ITGB4 enhanced KLF4 stability. **a-b** ITGB4 was knocked down in LN229 and U251 cells. The protein levels of ITGB4 and KLF4 were analysed by western blotting assay. **c** LN229 and U251 cells with or without ITGB4 knockdown were treated with MG132 or not. The protein levels of ITGB4 and KLF4 were analysed by western blotting assay. **d-g** LN229 cells with or without ITGB4 knockdown or overexpression were treated with CHX (10 mg/ml) for the indicated times. The half-life of KLF4 was measured. Data represent the mean ± SD of three independent experiments. ** *p* < 0.01, *** *p* < 0.001 vs. control. **h-i** LN229 cells with or without ITGB4 knockdown or overexpression, were transfected with the indicated constructs and the cells then were treated with MG132 for 8 h before collection. The whole-cell lysate was subjected to immunoprecipitation with anti-KLF4 antibodies and western blotted with anti-Ub antibodies to detect ubiquitylated KLF4. **j** LN229 and U251 cell lysates were subjected to immunoprecipitation with control IgG or anti-KLF4 antibodies. The immunoprecipitates were then detected using the indicated antibodies. **k-l** ITGB4 was knocked down or overexpressed in LN229 cells. The cell lysates were subject to immunoprecipitation with control IgG or anti-KLF4 antibodies. The immunoprecipitates were then detected using the indicated antibodies

**Fig. 8:**
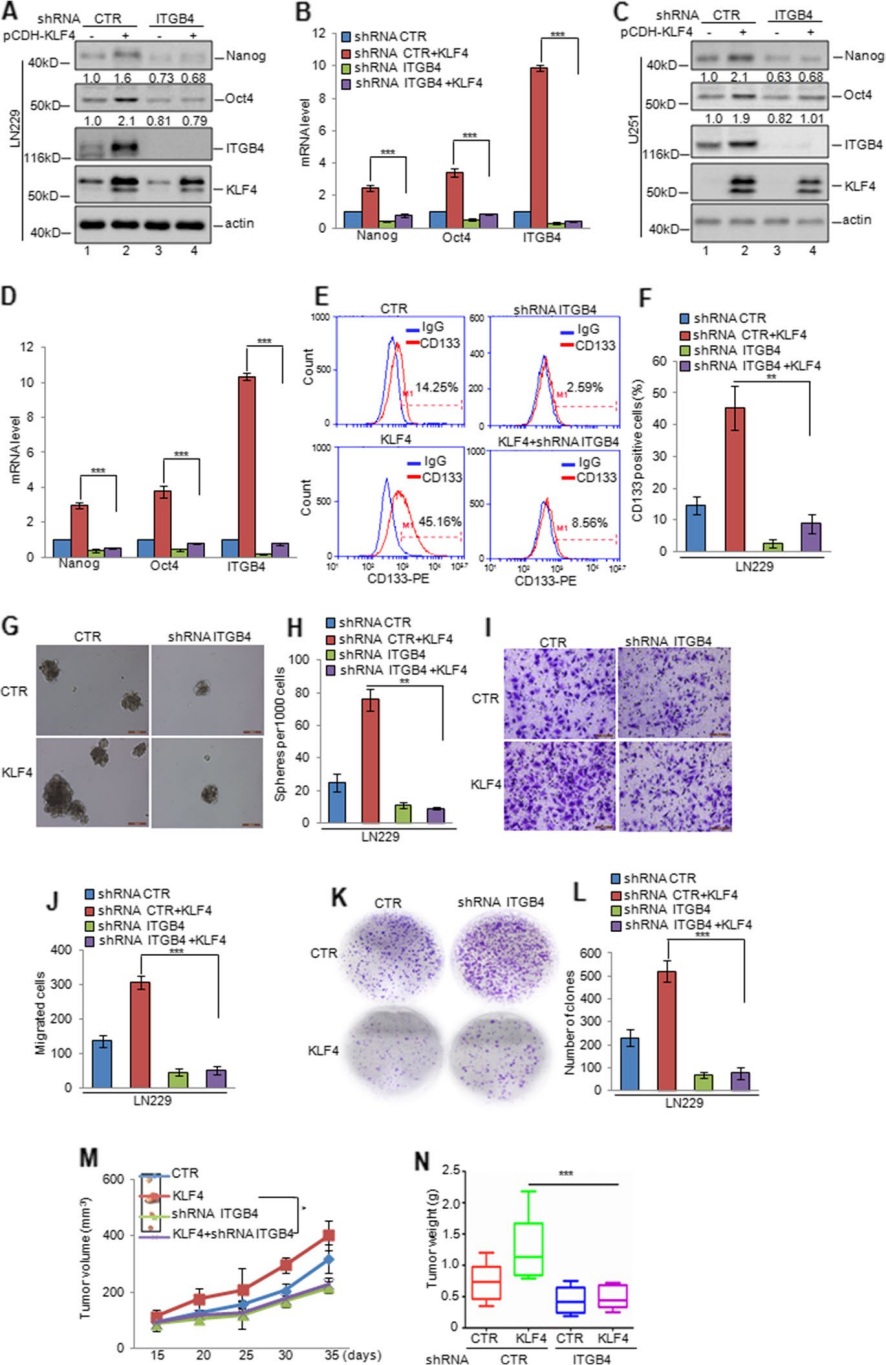
KLF4 and ITGB4 played an essential role in glioma tumorigenesis. **a-d** ITGB4 was knocked down in LN229 and U251 cells with or without overexpressing KLF4. The expression levels of ITGB4, Oct4, Nanog, and KLF4 were analysed by western blotting and q-RT-PCR assays. Data represent the mean ± SD of three independent experiments. *** *p* < 0.001 vs. control. **e–h** The CD133-positive populations and mammosphereforming abilities were analysed. Data represent the mean ± SD of three independent experiments. ** *p* < 0.01 vs. control. **i-l** Cell migration and proliferation were analysed by transwell and colony formation assays. Data represent the mean ± SD of three independent experiments. *** *p* < 0.001 vs. control. **m–n** The cells were subcutaneously injected into nude mice (*n* = 6 in each group) for tumour formation. Representative bright-field imaging of the tumours in the mice implanted with the indicated cells. After 5 weeks, mice receiving transplants of the indicated cells were sacrificed. The tumour volume and weight were calculated. ** *p* < 0.01, *** *p* < 0.001 vs. control

The corrections do not have any effect on the results or conclusions of the article.
