# Supplementary figures and images for: Reciprocal regulation of integrin β4 and KLF4 promotes gliomagenesis through maintaining cancer stem cell traits
Source: J Exp Clin Cancer Res. 2019 Jan 18;38:23. doi: 10.1186/s13046-019-1034-1 (PMC6339386; doi:10.1186/s13046-019-1034-1)

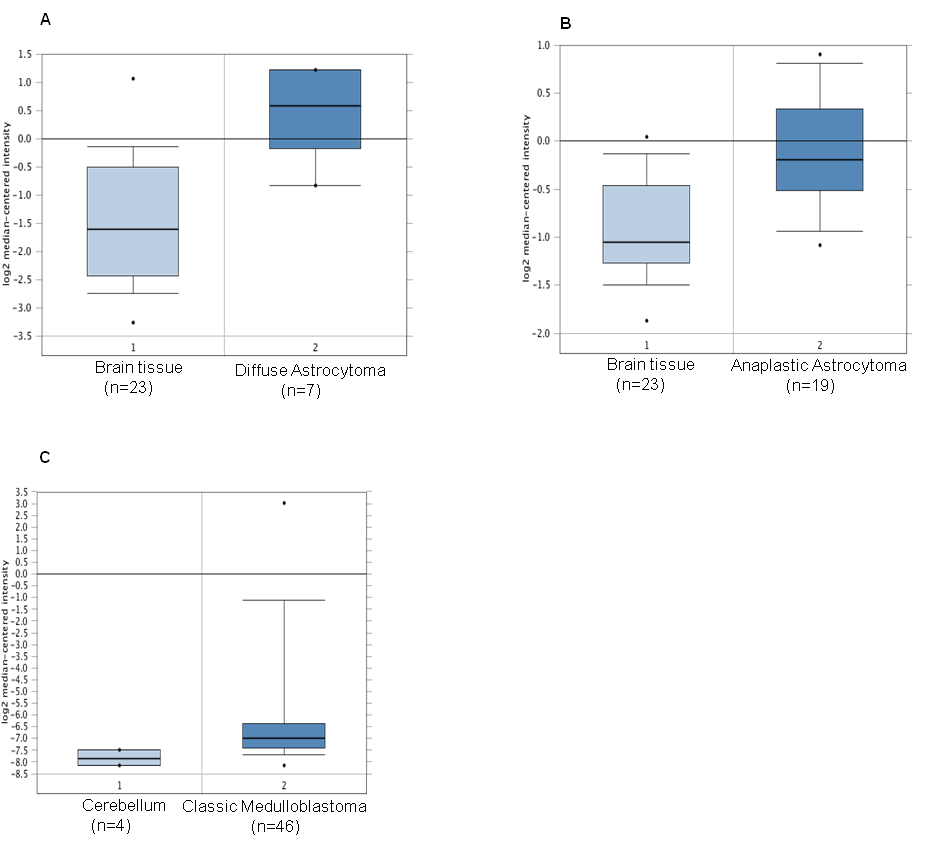

Supplement: Supplementary file 1 — Figure S1. (A) ITGB4 mRNA expression in brain tissues (n = 23) and diffuse astrocytoma tissues (n = 7) were analysed. (B) ITGB4 mRNA expression in brain tissues (n = 23) and anaplastic astrocytoma tissues (n = 19) were analysed. (C) ITGB4 mRNA expression in cerebellum (n = 4) and classic medulloblastoma (n = 23) were analysed. These data were extracted from the Oncomine database. (TIF 108 kb) [file 13046_2019_1034_MOESM1_ESM.tif]

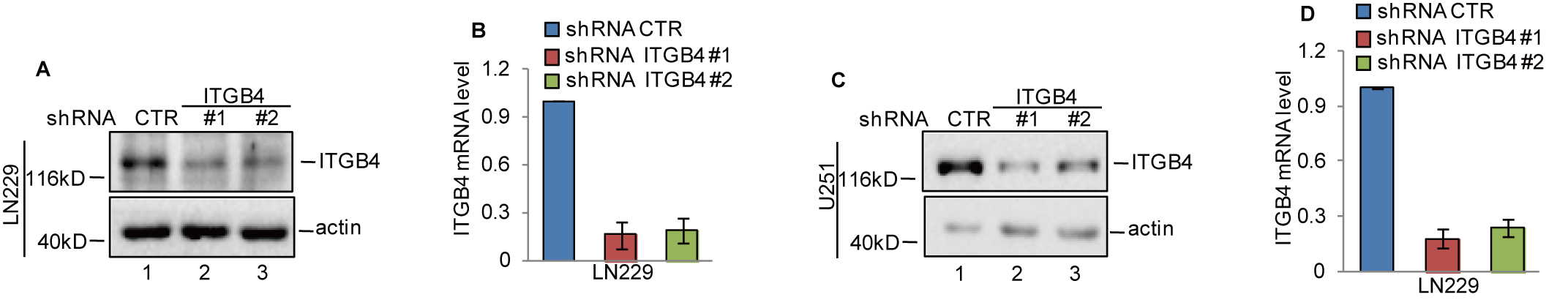

Supplement: Supplementary file 2 — Figure S2. (A-D) ITGB4 was knocked down in LN229 and U251 cells using shRNA. The expression levels of ITGB4 were analysed by western blotting and q-RT-PCR. (TIF 268 kb) [file 13046_2019_1034_MOESM2_ESM.tif]

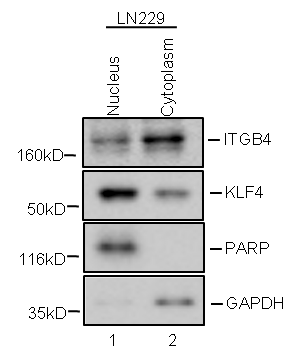

Supplement: Supplementary file 3 — Figure S3. nuclear and cytosol proteins were isolated from LN229 cells. The expression levels of ITGB4 and KLF4 were detected by western blotting. PARP was used as nuclear marker and GAPDH was used as cytoplasm marker. (TIF 39 kb) [file 13046_2019_1034_MOESM3_ESM.tif]
